# Activation of the TNF-α-Necroptosis Pathway in Parvalbumin-Expressing Interneurons of the Anterior Cingulate Cortex Contributes to Neuropathic Pain

**DOI:** 10.3390/ijms242015454

**Published:** 2023-10-22

**Authors:** Yiwen Duan, Qiaoyun Li, Yaohui Zhou, Shaoxia Chen, Yongyong Li, Ying Zang

**Affiliations:** 1 Guangdong Provincial Key Laboratory of Brain Function and Disease, Pain Research Center, Department of Physiology, Zhongshan Medical School, Sun Yat-Sen University, 74 Zhongshan Road 2, Guangzhou 510080, China; dyiwennn@163.com (Y.D.); liqy223@mail2.sysu.edu.cn (Q.L.); zhouyh93@mail2.sysu.edu.cn (Y.Z.); liyy@mail.sysu.edu.cn (Y.L.); 2 State Key Laboratory of Oncology in South China, Department of Anesthesiology, Sun Yat-Sen University Cancer Center, Collaborative Innovation Center for Cancer Medicine, 651 Dongfeng Road East, Guangzhou 510060, China; chenshx1@sysucc.org.cn

**Keywords:** neuropathic pain, anterior cingulate cortex, tumor necrosis factor-α, necroptosis, parvalbumin interneuron

## Abstract

The hyperexcitability of the anterior cingulate cortex (ACC) has been implicated in the development of chronic pain. As one of the key causes of ACC hyperexcitation, disinhibition of the ACC may be closely related to the dysfunction of inhibitory parvalbumin (PV)-expressing interneurons (PV-INs). However, the molecular mechanism underlying the ACC PV-INs injury remains unclear. The present study demonstrates that spared sciatic nerve injury (SNI) induces an imbalance in the excitation and inhibition (E/I) of the ACC. To test whether tumor necrosis factor-α (TNF-α) upregulation in the ACC after SNI activates necroptosis and participates in PV-INs damage, we performed a differential analysis of transcriptome sequencing using data from neuropathic pain models and found that the expression of genes key to the TNF-α-necroptosis pathway were upregulated. TNF-α immunoreactivity (IR) signals in the ACCs of SNI rats were co-located with p-RIP3- and PV-IR, or p-MLKL- and PV-IR signals. We then systematically detected the expression and cell localization of necroptosis-related proteins, including kinase RIP1, RIP3, MLKL, and their phosphorylated states, in the ACC of SNI rats. Except for RIP1 and MLKL, the levels of these proteins were significantly elevated in the contralateral ACC and mainly expressed in PV-INs. Blocking the ACC TNF-α-necroptosis pathway by microinjecting TNF-α neutralizing antibody or using an siRNA knockdown to block expression of MLKL in the ACC alleviated SNI-induced pain hypersensitivity and inhibited the upregulation of TNF-α and p-MLKL. Targeting TNF-α-triggered necroptosis within ACC PV-INs may help to correct PV-INs injury and E/I imbalance in the ACC in neuropathic pain.

## 1. Introduction

The anterior cingulate cortex (ACC) is a brain region key to mediating pain perception and emotional responses to it, including pain aversion and empathy, through the descending facilitation of spinal nociceptive transmission and communication with brain regions associated with emotional and motivational processing [1,2,3,4,5,6,7]. The ACC contains multi-layer pyramidal cells innervated by the excitatory fibers of pyramidal neurons within other layers of the ACC or other brain regions receiving sensory input [8,9]. Furthermore, a large number of interneurons span across ACC pyramidal neurons, most of which are inhibitory neurons that contain gamma-aminobutyric acid (GABA) or neuropeptides. Within these local circuits, inhibitory neurons receive glutamate excitatory activation from pyramidal neurons and regulate pyramidal cell activity through GABA inhibitory synapses to maintain the balance between excitation and inhibition (E/I) [8]. The disruption of this dynamic E/I balance due to impaired inhibitory GABAergic neurotransmission may induce chronic pain [10].

Extensive evidence implicates ACC hyperexcitation in the development of chronic pain and pain-related negative emotions [1,5,6,7,11,12]. As one of the key causes of ACC hyperexcitation, disinhibition of the ACC may be related to the dysfunction of inhibitory parvalbumin (PV)-expressing interneurons (PV-INs). Although the numbers of PV-INs and somatostatin-positive inhibitory interneurons were observed at significantly low levels in a chronic inflammatory pain model of ACC [13], activation of PV-INs but not somatostatin-positive interneurons using optogenetic or chemogenetic techniques to compensate for the diminished inhibitory activity reduces peripheral mechanical hypersensitivity and alleviates pain-anxiety-like behaviors [13,14], indicating the important role that PV-INs play in pain modulation. Furthermore, peripheral nerve injury induced by spared sciatic nerve injury (SNI) also reduces the number of ACC PV-INs by inducing activated microglia to phagocytose PV-INs [15]. At present, however, the molecular mechanism underlying the structural and functional impairment of ACC PV-INs under the condition of chronic pain remains unclear.

A key factor in the initiation and maintenance of neuropathic pain, tumor necrosis factor α (TNF-α), is over-expressed in peripheral afferents, the spinal cord, and brain regions associated with pain processing, as well as in the ACC following peripheral nerve injury [6,16,17,18,19]. Neutralizing up-regulated TNF-α by microinjecting anti-TNF-α antibody into the ACC blocks the hyperexcitation of neurons within it and alleviates SNI-induced pain aversion and mechanical allodynia [6]. Identified in 2018 [20], necroptosis is a mode of TNF-α-mediated programmed cell death that combines the amenability of apoptosis to regulation with the morphological characteristics of cell necrosis [21,22,23,24]. Recent studies have shown that chronic constriction injury (CCI) to the sciatic nerve, a classic model of pain, increases the expressions of inflammation factor TNF-α and its downstream necroptosis-related proteins (serine-threonine kinases receptor-interacting protein 1 [RIP1] and 3 [RIP3]) in the DRG, spinal cord, and hippocampus. Further, elevated levels of programmed cell death protein 1 after CCI indicate cell death in peripheral and central nervous tissues [25]. However, the literature features no report of whether the ACC is damaged by necroptosis under conditions of chronic pain, what its related cell phenotypes are, or what role it plays in pain modulation.

The present study examines the activation of the necroptotic pathway downstream of TNF-α in the ACC after SNI and its association with PV-IN damage. Our results show that SNI enhances the expression of PV-IN necroptosis-related proteins, including phosphorylated RIP1 (p-RIP1), RIP3, p-RIP3, and phosphorylated mixed lineage kinase domain-like protein (p-MLKL) in the ACC. We further found that neutralizing the up-regulated TNF-α by microinjecting anti-TNF-α antibody in the ACC or using small interfering RNA (siRNA) to knockdown MLKL expression in the ACC inhibits SNI-induced mechanical allodynia via blocking the TNF-α-necropotosis pathway. Finally, the present investigation reveals the molecular mechanism underlying the E/I imbalance mediated by impaired PV-INs within the ACC after SNI. These findings may help to elucidate the pathogenetic mechanism of chronic pain and inform novel clinical treatments.

## 2. Results

### 2.1. SNI Induces Excitation/Inhibition (E/I) Imbalance in ACC

**Figure 1:**
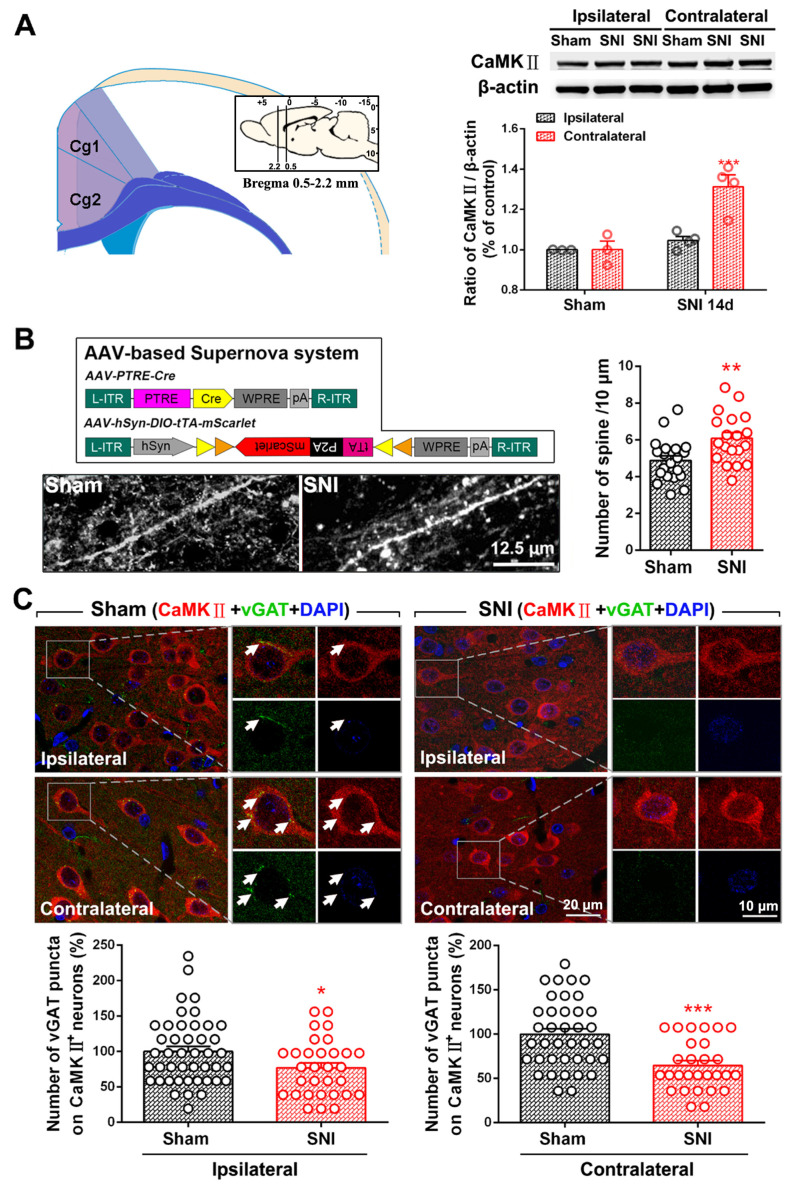
SNI induces an excitation/inhibition (E/I) imbalance in the ACC neural network structure. (**A**) SNI-induced upregulation of CaMKⅡ (a pyramidal neuron marker) in the contralateral ACC. The left part of the image shows that the ACC samples used to perform the Western blot include the Cg1 and Cg2 regions located between 2.2 and 0.5 mm on the anterior coronal plane of the bregma. A representative Western blot of CaMKⅡ expression in the bilateral ACC and the quantitative results of the Western blot is shown on the right. *** *p* < 0.001 versus the sham group (two-way ANOVA). (**B**) Supernova revealed a significant increase in the number of dendritic spines of CaMKⅡ-positive neurons in the ACC of mice subjected to SNI. Upper left: schematic for the Supernova vector set for sparsely labeled ACC neurons. Representative data and quantitative analysis are shown on the lower left and right, respectively. Scale bar = 12.5 μm. ** *p* < 0.01 versus the sham (unpaired *t* test). (**C**) SNI reduces inhibitory synaptic terminals on the surface of bilateral ACC pyramidal neurons. Upper, representative double immunofluorescence staining image showing CaMKⅡ-positive (red) somatic vGAT puncta-IR (inhibitory synaptic terminal marker, green) in the sham and SNI groups in the bilateral ACC. An enlarged image of the area enclosed by the white box is shown on the right. The white arrow indicates the colocalization (yellow) of vGAT puncta with CaMKⅡ. Blue fluorescence corresponds to DAPI, a nuclear counterstain. Below is the quantitative analysis of the number of vGAT-IR puncta on CaMKⅡ-positive neurons in the bilateral ACC of the sham and SNI groups. * *p* < 0.05, *** *p* < 0.001 versus the sham (unpaired *t* test).

Our previous study showed that the number of PV-INs and inhibitory synaptic terminals is significantly reduced in the bilateral ACC of rats with SNI-induced chronic pain [15]. To determine whether SNI affects the E/I balance in the ACC, we assessed the expression of the pyramidal neuron marker CaMKⅡ, changes in the dendritic spine density of glutaminergic CaMKⅡ immunoreactivity (IR) neurons, and changes in inhibitory synaptic terminals acting on pyramidal neurons after subjecting the rats to SNI. We found that the level of CaMKⅡ on the contralateral side of the ACC, but not the ipsilateral side, significantly increased on PO day 7 (*p* < 0.001, Figure 1A). Using the Supernova method, we further observed a significant increase in the number of dendritic spines of CaMKⅡ-positive neurons in the ACC of mice subjected to SNI (*p* < 0.01, Figure 1B). This finding suggests that SNI enhances the activity of ACC pyramidal neurons. Furthermore, as indicated by a decrease in vGAT-IR puncta on the surfaces of CaMKⅡ-positive neurons, SNI decreases the innervation of bilateral ACC pyramidal neurons by inhibiting synaptic terminals (*p* < 0.05 ipsilaterally, *p* < 0.001 contralaterally, Figure 1C). These results suggest that SNI induces an E/I imbalance in the bilateral ACC neural network, i.e., SNI weakens inhibitory synaptic connections and strengthens excitatory neuronal output.

### 2.2. The Activation of the TNF-α-Necroptosis Pathway in ACC PV-INs Following SNI Surgery

**Figure 2:**
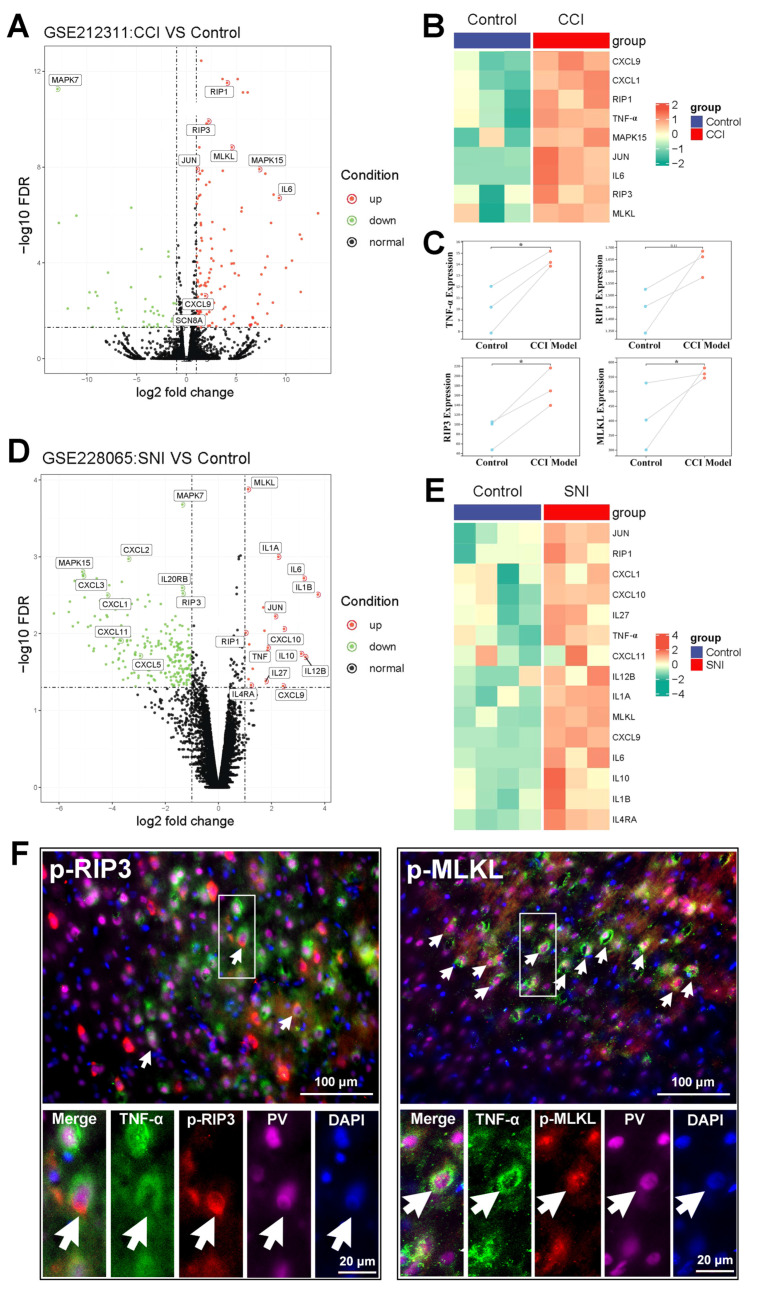
TNF-α-necroptosis pathway is activated in the ACC of neuropathic pain models. Volcano map (**A**) and heat map (**B**) showing the expression of *TNF-α*, *RIP1*, *RIP3*, and *MLKL* in the ACC of CCI model of neuropathic pain (*n* = 3/group) and controls (*n* = 3/group) (data from GEO dataset GSE212311). The Hub gene expression data of CCI model (red dots) and control (green dots) for *TNF-α*, *RIP1*, *RIP3*, and *MLKL* are shown in (**C**). * *p* < 0.05 versus the control (unpaired *t* test). Volcano map (**D**) and heat map (**E**) showing the expression of *TNF-α*, *IL-6*, *RIP1*, and *MLKL* in the ACC of SNI model (*n* = 3/group) and controls (*n* = 3/group) (data from GEO dataset GSE228065). (**F**) Representative triple staining shows the overlap of TNF-α (green) with p-RIP3 (red) and PV (magenta), or p-MLKL (red) and PV (magenta) on PO day 7. Enlarged and color-split images of the area enclosed in white boxes are shown below. White arrows indicate co-localization. Blue fluorescence corresponds to DAPI.

To determine whether the TNF-α-necroptosis pathway is activated in the ACC following peripheral nerve injury, a differential analysis of transcriptome sequencing was performed using data from neuropathic pain models (GEO datasets GSE212311 and GSE228065). The expression of genes key to the TNF-α-necroptosis pathway, including *TNF-α*, *RIP3*, and *MLKL*, was enhanced to a greater degree in the ACC of the CCI model than in the control group (*p* < 0.05, Figure 2A–C). The expression of *TNF-α*, *RIP1*, and *MLKL* was enhanced in the SNI model (Figure 2D,E). The present immunohistochemical triple staining study revealed that TNF-α-IR signals in the ACC of rats subjected to SNI were co-located with p-RIP3- and PV-IR signals, or p-MLKL- and PV-IR signals (Figure 2F), suggesting that SNI may activate the TNF-α-necroptosis pathway in PV-INs, induce PV-IN injury, and cause functional decline.

**Figure 3:**
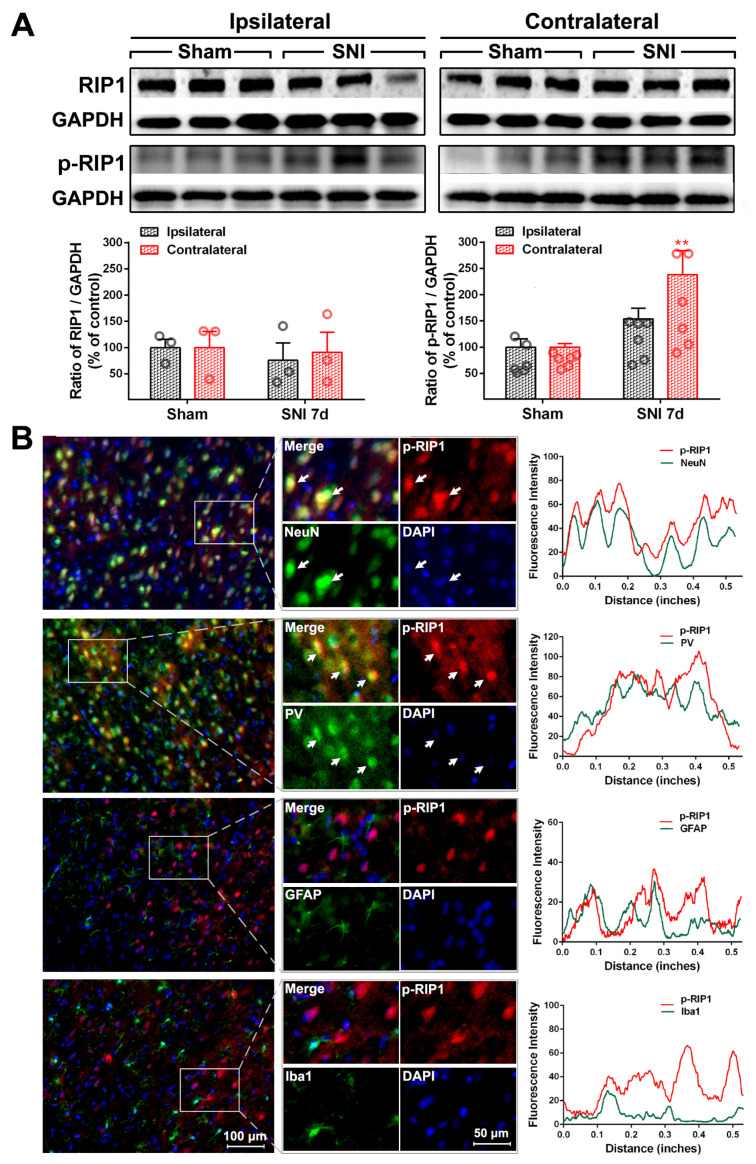
SNI increases the expression of necroptosis-related protein p-RIP1 in the contralateral ACC (**A**) Representative Western blot of RIP1 and p-RIP1 expression in the bilateral ACC is shown in the top panel. The quantitative results of Western blotting protein are shown below. SNI induces the expression of p-RIP1in the contralateral ACC at postoperative (PO) day 7. ** *p* < 0.01 versus the sham group (two-way ANOVA). (**B**) Representative double staining shows the overlap (yellow) of p-RIP1 (red) with NeuN (neuronal marker, green) and PV (PV-IN marker, green), but not with GFAP (astrocyte marker, green) or Iba1 (microglia marker, green), on PO day 7. Enlarged and color-split images of the area enclosed in white boxes are shown in the middle. White arrows indicate co-localization (yellow). Blue fluorescence corresponds to DAPI. The fluorescence intensity curves for red and green from boxed areas are shown on the right side of each group.

**Figure 4:**
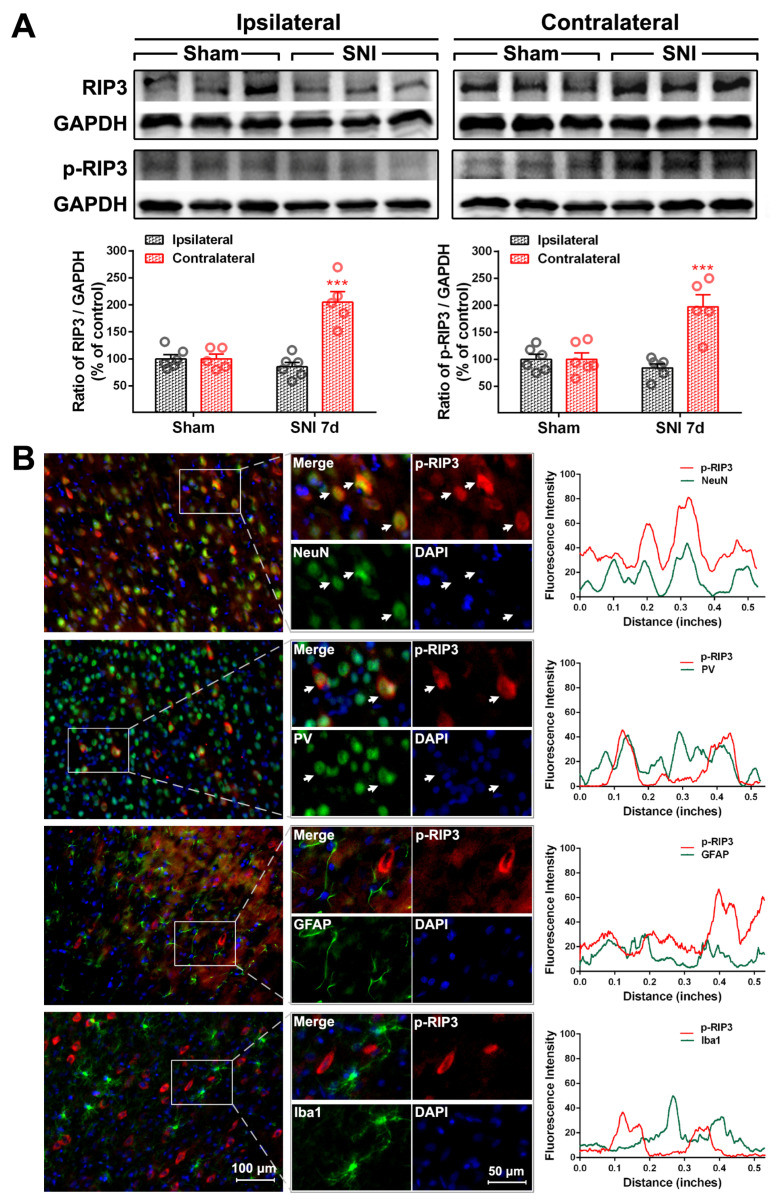
SNI upregulates RIP3/p-RIP3 proteins in the contralateral ACC (**A**) Representative Western blot of RIP3 and p-RIP3 expression in the bilateral ACC is shown in the top panel. Quantitative results of Western blot are shown below. SNI upregulates RIP3 and p-RIP3 in the contralateral ACC on PO day 7. *** *p* < 0.001 versus the sham group (two-way ANOVA). (**B**) Representative double staining showing the overlap (yellow) of p-RIP3 (red) with NeuN (green) and PV (green), but not with GFAP (green) or Iba1 (green), on PO day 7. Enlarged and color-split images of areas enclosed in white boxes are shown in the middle. White arrows indicate co-localization (yellow). Blue fluorescence corresponds to DAPI. The fluorescence intensity curves for red and green from boxed areas are shown on the right side of each group.

**Figure 5:**
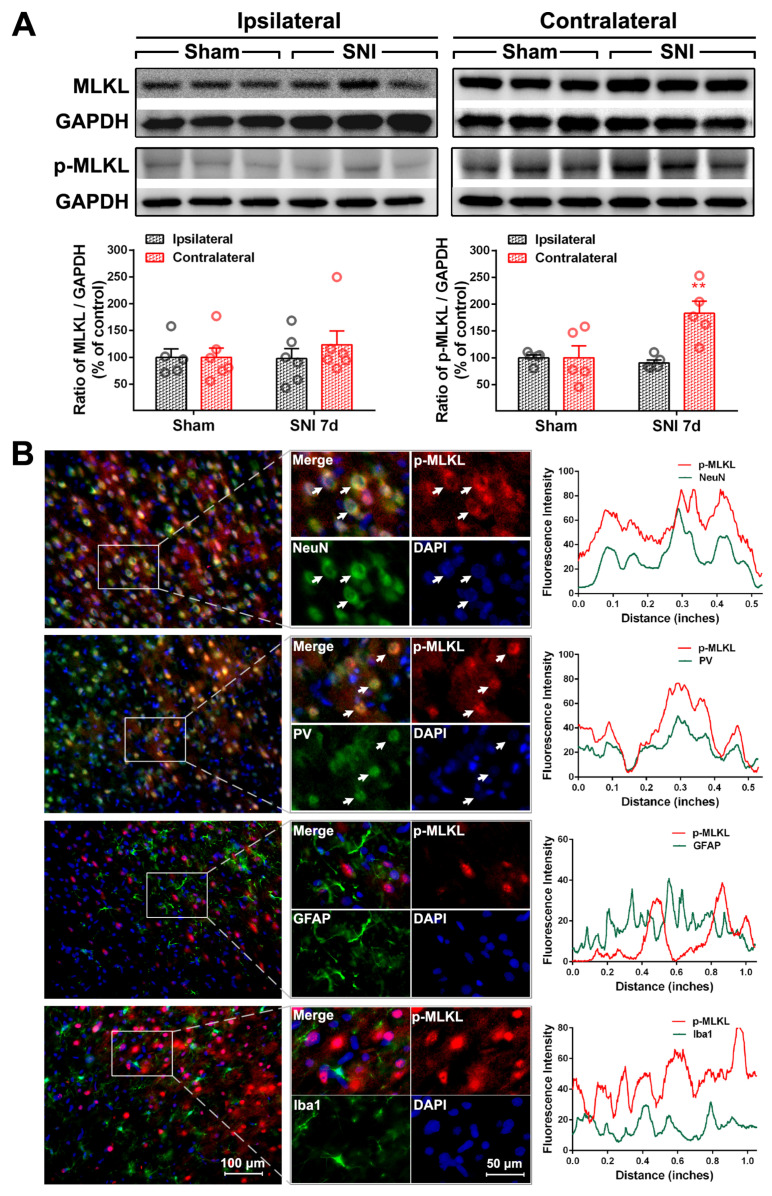
SNI increases the expression of necrotizing membrane-destroying proteins p-MLKL in the contralateral ACC (**A**) Representative Western blot of MLKL and p-MLKL expression in the bilateral ACC is shown in the top panel. Quantitative results of Western blot are shown below. SNI upregulates p-MLKL in the contralateral ACC on PO day 7. ** *p* < 0.01 versus the sham group (two-way ANOVA). (**B**) Representative double staining showing the overlap (yellow) of p-MLKL (red) with NeuN (green) and PV (green), but not with GFAP (green) or Iba1 (green), on PO day 7. Enlarged and color-split images of areas enclosed in white boxes are shown in the middle. White arrows indicate co-localization (yellow). Blue fluorescence corresponds to DAPI. The fluorescence intensity curves for red and green from boxed areas are shown on the right side of each group.

To explore whether the E/I imbalance in the ACC following SNI is related to the necroptosis of PV-INs, we examined the expression and cell localization of necroptosis-related proteins in the ACC of SNI rats. Relative to the sham group, the protein levels of p-RIP1 (*p* < 0.01, Figure 3A), RIP3, p-RIP3 (*p* < 0.001, Figure 4A), and p-MLKL (*p* < 0.01, Figure 5A) in the contralateral ACC were significantly elevated on PO day 7. The protein expressions of RIP1 and MLKL did not increase in both sides of the ACC, and there was no statistically significant difference between the sham group and the SNI group. Immunofluorescence double-staining cell localization analysis showed that p-RIP1 (Figure 3B and Table 1), RIP3 (Supplementary Figure S2 and Table 1), p-RIP3 (Figure 4B and Table 1), MLKL (Supplementary Figure S3 and Table 1), and p-MLKL (Figure 5B and Table 1) were mainly expressed in NeuN-IR and PV-IR neurons; principally observed in astrocytes, this observation did not apply to RIP1 expression (Supplementary Figure S1 and Table 1).

### 2.3. Inhibition of the ACC TNF-α-Necroptosis Pathway Reduces Neuroinflammation in the ACC and Alleviates SNI-Induced Mechanical Allodynia

**Figure 6:**
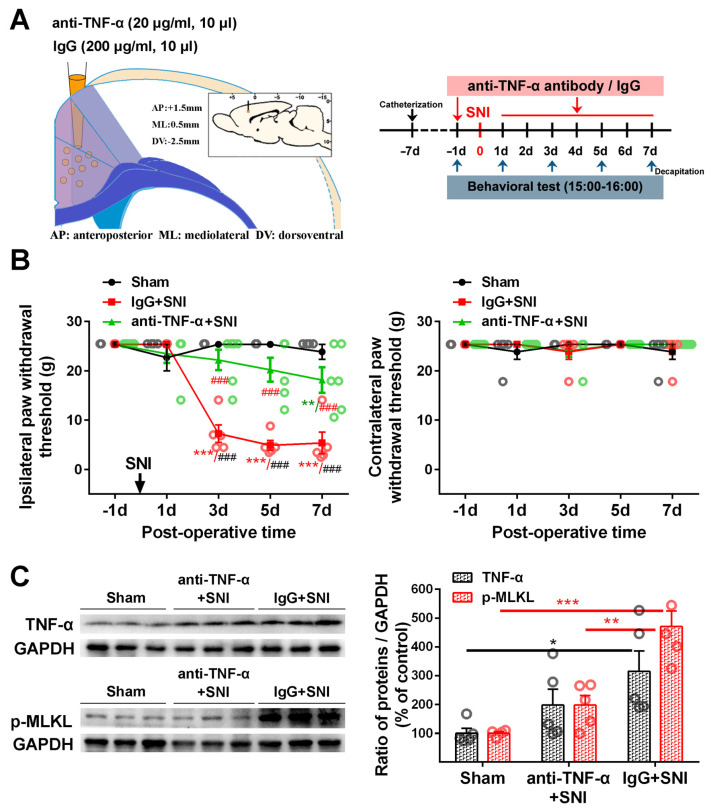
The effect of neutralizing SNI-induced elevation of TNF-α in the ACC on pain behavior and necroptosis activation (**A**) The injection site of anti-TNF-α antibodies (20 μg/mL, 10 μL) or control IgG (20 μg/mL, 10 μL) in the contralateral ACC is shown on the left, and behavioral test paradigms are shown on the right. (**B**) Changes in the bilateral paw withdrawal thresholds (PWTs) in the sham, IgG+SNI, and anti-TNF-α+SNI groups. SNI-induced ipsilateral mechanical allodynia in the vehicle control group could be significantly mitigated by microinjecting a TNF-α neutralization antibody on postoperative (PO) days 3, 5, and 7. ** *p*-value < 0.01, *** *p*-value < 0.001 versus PO day − 1 (Dunn’s multiple comparisons test) or ### *p*-value < 0.001 comparison among groups (multiple *t* test). (**C**) Representative Western blot showing the effect of the TNF-α neutralization antibody on the expression of TNF-α and p-MLKL in the contralateral ACC (left). Protein quantification results are shown right. * *p*-value < 0.05, ** *p*-value < 0.01, *** *p*-value < 0.001 (two-way ANOVA).

Similar to what we observed in our previous study [6], microinjecting TNF-α neutralization antibody into the contralateral ACC 1 day before SNI and again on each of the 7 days following SNI (Figure 6A) could significantly mitigate SNI-induced ipsilateral mechanical allodynia (Figure 6B). In the vehicle IgG control + SNI group, the SNI-induced mechanical withdrawal threshold of the injured hind paw decreased significantly from PO day 3 to the end of the observation period on PO day 7 (*p* < 0.001). Compared with the vehicle control, the ipsilateral paw withdrawal threshold (PWT) in the anti-TNF-α+SNI group was significantly elevated (*p* < 0.001). There was no significant difference between the anti-TNF-α+SNI and sham groups or across the surgical intervention on PO days 3 and 5 (Figure 6B). The Western blot showed that the SNI-induced overexpression of TNF-α and p-MLKL in the ACC was inhibited by microinjecting TNF-α neutralizing antibodies into the ACC (Figure 6C), implicating the blockage of the necroptosis pathway and the inhibition of ACC TNF-α in the central negative regulation of chronic pain.

**Figure 7:**
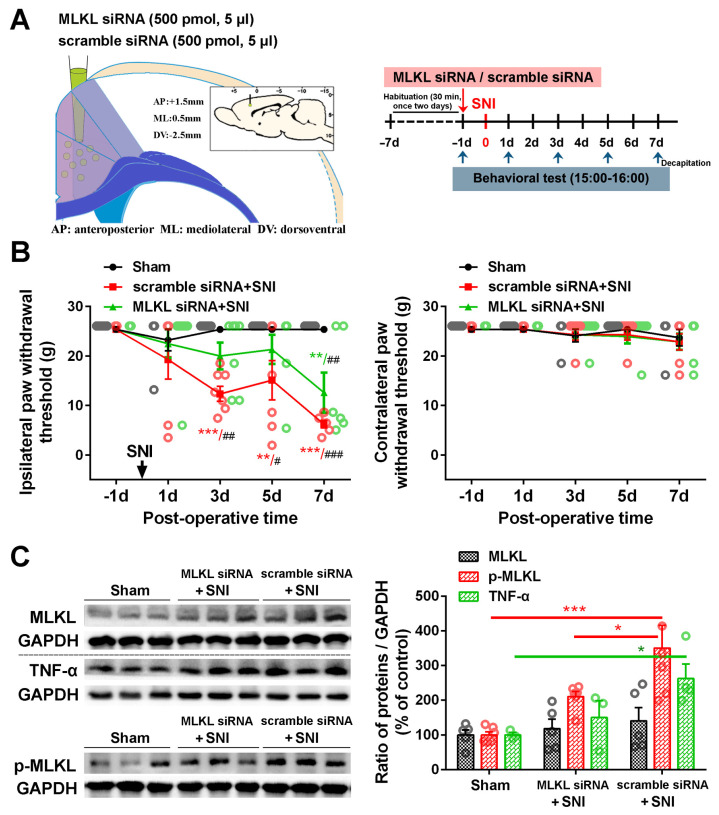
The effect of MLKL knockdown in the contralateral ACC on pain behavior and TNF-α expression. (**A**) The injection site of MLKL siRNA or control siRNA in the contralateral ACC is shown on the left, and the behavioral test paradigms are shown on the right. (**B**) Changes in the bilateral paw withdrawal thresholds (PWTs) in the sham, control siRNA+SNI, and MLKL siRNA+SNI groups. SNI-induced ipsilateral mechanical allodynia in the control siRNA group could be partially blocked by MLKL siRNA on postoperative (PO) days 3 and 5. ** *p*-value < 0.01, *** *p*-value < 0.001 versus PO day 1 (Dunn’s multiple comparisons test) or # *p*-value < 0.05, ## *p*-value < 0.01, ### *p*-value < 0.001 comparison among groups (multiple *t* test). (**C**) Representative Western blot showing the effect of the MLKL siRNA on the expression of MLKL, p-MLKL, and TNF-α in the contralateral ACC (left). Protein quantification results are shown right. * *p*-value < 0.05, *** *p*-value < 0.001 (two-way ANOVA).

To further determine the role of the ACC necroptosis pathway in neuropathic pain, we used a siRNA knockdown to block the expression of MLKL in the contralateral ACC. We found that MLKL siRNA could partially alleviate the decrease in PWT induced by SNI. In contrast to the significant decrease in PWT on PO day 3 in the control siRNA group, the drop in PWT started on PO day 7 in the MLKL siRNA group (Figure 7B). Western blot confirmed that MLKL siRNA effectively reduced the levels of p-MLKL and TNF-α in the contralateral ACC (Figure 7C). These results suggest that interfering with the activation of the necroptotic pathway in the ACC can inhibit neuroinflammation and delay the induction of chronic pain after a nerve injury.

## 3. Discussion

This study demonstrated that SNI induces an E/I imbalance in the ACC. Analyzing the genetic expression of proteins key to the TNF-α-necroptosis pathway in CCI and SNI neuropathic pain models, we detected the expression and cell localization of necroptosis-related proteins, including RIP1/p-RIP1, RIP3/p-RIP3, and MLKL/p-MLKL, in the ACC of SNI rats. Specifically, we found that the levels of these proteins (with the exception of RIP1 and MLKL) were significantly elevated in the contralateral ACC and mainly expressed in PV-INs. Neutralizing the up-regulated TNF-α by microinjecting anti-TNF-α antibody into the ACC or using siRNA to knockdown MLKL expression in the ACC effectively alleviated pain hypersensitivity and blocked the SNI-induced upregulation of TNF-α and necroptosis-related proteins. Necroptosis triggered by TNF-α in the PV-INs of the ACC may be a key contributor to the E/I imbalance in the ACC and neuroimmune responses following peripheral nerve injury.

### 3.1. ACC Hyper-Excitability Induces Pain Hypersensitivity and Pain-Related Emotional Disorders

Numerous neuroimaging studies have confirmed that peripheral nerve injury can lead to reorganization of the central nervous system, including the cerebral cortex, relay nucleus, thalamus, brainstem, and spinal cord, while corresponding brain area function decreases [26,27,28]. A neuroimaging meta-analysis showed that the ACC was one of the most consistently activated neural regions in patients with chronic pain [29]. Reflecting these clinical data, animal models of neuropathic pain induced by CCI and SNI exhibited increased excitability of pyramidal neurons in lamina 5 of the ACC [10,30]. In addition to changes in intrinsic excitability, the synaptic input from dominating neural networks can alter the activity of neurons. Peripheral nerve injury can induce the long-term potentiation (LTP) of ACC excitatory synaptic transmission [8,31,32]. The confluence of two major forms of LTP in ACC excitatory synapses, namely NMDA receptor-dependent postsynaptic LTP and NMDA receptor-independent presynaptic LTP, may contribute to the synaptic mechanism underlying the involvement of the ACC in chronic pain and pain-related emotional disorders [33,34].

The hyper-excitability of the ACC can trigger changes in spinal networks [35], thus facilitating spinal nociceptive transmission through the periaqueductal gray (PAG) and rostral ventral medulla (RVM) or through the ACC’s direct corticospinal projections [3,4,36,37,38]. Abnormal neuroimmune activity in the ACC caused by peripheral nerve injury may also exacerbate spinal cord neuroinflammation and, consequently, nociceptive sensitization by promoting corticospinal projections [5]. Furthermore, the ACC sends extensive projections to other limbic regions that regulate emotional and motivational states, such as the thalamus, insula, amygdala, and nucleus accumbens (NAc) [2,39]. The ACC is also, therefore, an important center for processing and evaluating the emotional components of pain [40,41,42].

### 3.2. Dysfunction of ACC Inhibitory Interneurons in Chronic Pain Promotes E/I Imbalance in the ACC

As the activity of neurons is influenced by the neural network in which they are situated, the increased excitability of ACC pyramidal neurons in chronic pain is related to the dysfunction of afferent inhibitory synapses. Both the connection of inhibitory synapses to excitatory pyramidal neurons and the innervation of inhibitory interneurons by excitatory neurons are significantly diminished in the CCI-induced animal model of pain; their reduced activity eventually results in the disinhibition of the ACC [10]. In a model of chronic inflammatory pain, the complete Freund’s adjuvant reduced vesicular GABA transporter expression at presynaptic terminals in the ACC without changing protein levels of GABAA receptor subunits and diminished the frequency of miniature and spontaneous inhibitory postsynaptic currents [43]. By contrast, decreasing the activity of the ACC network by inhibiting ACC pyramidal neurons and stimulating the Gs-coupled dopamine-D1 receptor to open the hyperpolarization-activated cyclic nucleotide-gated (HCN) channels significantly lessens input resistance and excitability, thus attenuating nociceptive responses and negative emotional behaviors in animal models of neuropathic pain [1]. Alternatively, the same effect can be achieved by stimulating ACC interneurons with oxytocin, thus enhancing inhibitory transmission [41]. Consistent with the results of animal studies, magnetic resonance spectroscopy analysis of patients with migraine revealed a positive correlation between increased ACC GABA levels and improved clinical outcomes [44].

In a previous study, we showed that SNI significantly reduces the number of inhibitory PV-INs and vGAT-positive inhibitory synaptic terminals in the ACC but not the number of excitatory neurons and induces the microglial phagocytosis of inhibitory synapses [15]. This study expands upon these findings by confirming that SNI increases the expression of excitatory CaMKII protein and dendritic spine density of CaMKII-positive neurons in the ACC and decreases the density of inhibitory synaptic terminals wrapped around CaMKII-positive neurons (Figure 1). These observations suggest that SNI promotes the restructuring of the ACC nerves and weakens the inhibitory innervation of excitatory pyramidal neurons. Disinhibition of the ACC may account for ACC hyper-excitability and promote spinal cord nociceptive signaling.

### 3.3. The Role of the TNF-α-Necroptosis Pathway in ACC Disinhibition Following Peripheral Nerve Injury

Peripheral nerve injury can lead to inflammation and cell apoptosis of the nervous system in patients, including the prefrontal cortex, hippocampus, amygdala, thalamus, and periaqueductal gray matter [45,46,47,48]. The classic SNI sciatica model validates these findings, further confirming the destruction of CNS structures in neuropathic pain due to peripheral nerve injury [49]. As an important promoter of neuronal inflammation and modulator of neuronal excitability, TNF-α regulates voltage-gated sodium channels to sensitize primary nociceptive afferents [17,50]. It also mediates spinal and supraspinal E/I imbalances by enhancing excitatory and reducing inhibitory synaptic transmissions [51,52,53,54,55]. Our previous studies have shown that the overexpression of TNF-α in the ACC may promote the hyper-excitability of the ACC through the elevated voltage-gated sodium channel Nav1.6, which plays an important role in the regulation of pain transduction and aversion [5,6]. However, whether it can regulate the inhibitory function of the ACC neural network remains unclear.

Necroptosis induced by the binding of TNF-α to its receptor TNFR1 can cause the release of intracellular contents and thus trigger inflammation [22,56,57,58,59]. As two essential components in necrosome assembly, RIP1 and RIP3 kinases play a central role in TNF-α-induced programmed necrosis [23]. During the onset of necroptosis, the autophosphorylation of RIP1 is required for the homologous oligomerization of RIP3 [60]. Subsequently, p-RIP3 phosphorylates MLKL to form oligomers bound to phosphatidylinositol lipids and cardiolipids; p-MLKL then moves to the plasma membrane, destroys its integrity, and initiates necroptosis [61,62]. RIP3 reportedly promotes the secretion of pro-inflammatory cytokines TNF-α and interleukin-1β by activating inflammasome 3 and caspase-1 [63], thus recruiting, activating, and transforming microglia [57]. Blocking the MAPK/NF-κB pathway of activated microglia can inhibit the production of inflammatory factors such as TNF-α and thus prevent microglia-mediated neuronal necroptosis [64]. Therefore, a positive pro-inflammatory feedback loop could exist between TNF-α and necroptosis that can trigger neuroinflammation and promote neuropathic pain [57,59,65,66,67]. Several lines of evidence suggest that necroptosis-related proteins in peripheral sensory afferent neurons and the spinal cord contribute to chronic pain [68,69,70,71,72,73]. However, the activation and role of the TNF-α-necroptosis pathway in the cortex in pain-related disorders have received limited attention [5].

To confirm whether necroptosis signals in the ACC region are involved in chronic pain, we performed a Gene Set Enrichment Analysis (GSEA) using data obtained from two animal models of chronic pain (GSE212311 and GSE228065). We found that the expression of proteins associated with the TNF-α/necrosome/MLKL axis was significantly higher in the ACC of animals with chronic pain than in healthy controls (Figure 2A–E), implicating the cortical TNF-α-necroptosis pathway in the progression of chronic pain. We further observed elevated concentrations of proteins related to the TNF-α-necroptosis pathway, including p-RIP1, RIP3, p-RIP3, and p-MLKL, in the contralateral ACCs of rats with SNI-induced chronic pain (Figure 3A, Figure 4A and Figure 5A). Double immunofluorescence staining identified SNI-induced necroptosis-related proteins in the ACC co-localizing mainly with PV-IR signals (Figure 3B, Figure 4B and Figure 5B, Supplementary Figures S1–S3, Table 1). The overexpression of TNF-α in the ACC following SNI may constitute a predisposing factor for the activation of the necroptotic pathway in PV-INs [6]. Immunohistochemical triple staining showed that TNF-α-IR colocalized with the p-RIP3- or p-MLKL-IR signals on PV-IR cells (Figure 2F). Neutralizing SNI-induced elevated levels of TNF-α by microinjecting anti-TNF-α antibodies into the contralateral ACC not only alleviated SNI-induced mechanical allodynia but also blocked the necroptosis of ACC PV-Ins (Figure 6). Further studies showed that the siRNA knockdown of the expression of MLKL in the ACC significantly alleviated mechanical allodynia in rats (Figure 7). This finding suggests that negative regulation of the TNF-α-necroptotic pathway in the ACC and the correction of its E/I imbalance may inform a new strategy for the clinical treatment of chronic pain.

## 4. Materials and Methods

### 4.1. Animals

The adult male Sprague-Dawley rats (150–200 g) and C57BL/6 mice (20–30 g) used in the present study were housed in individual cages. Ambient humidity (50–60%), a temperature of 24 °C, and a 12-h light/dark cycle (6 a.m.–6 p.m.) were rigorously maintained. Food and water were offered free of charge. The animals were evenly divided into three groups, namely the medication group, solvent group, and sham surgery group, with a total of 6–10 animals in each group, while maintaining the same conditions.

### 4.2. Spared Nerve Injury (SNI)

The SNI operation was performed as previously described [5,6]. By intraperitoneal injection (i.p.), anesthesia was administered at a concentration of 0.4% pentobarbital sodium and a dose of 40mg/kg (Sigma Aldrich, Taufkirchen, Germany), followed by skin lamination on the outside surface of the left hind thigh to reveal a fungal lesion and its three terminal branches: the tibial nerve, sural nerve, and common peroneum nerve. Finally, we tied the peroneus nerve and the tibia nerve and cut 2 mm from their ends to prevent damage to the sural nerve. For comparison, the same exposure procedure was performed for intact nerves in the sham surgery group.

### 4.3. Fifty Percent Paw Withdrawal Threshold Test

The mechanical allodynia of rat hindlimbs was measured with the Von Frey hair up-down method [5,6]. Briefly, animals were placed in an isolated Plexiglas compartment on a grid board. After habituating the tested animals for 15 min, we performed an allodynia test. Von Frey’s special cilia used for testing (0.41, 0.70, 1.20, 2.04, 3.63, 5.50, 8.51, and 15.14 g; where stiffness was increased logarithmically) were placed bilaterally on both sides of the rear paws, starting at 2.04 g. We recorded the fifty percent paw withdrawal threshold, and the response to mechanical stimulation at different post-operative (PO) times was assessed.

### 4.4. Immunohistochemistry and Immunofluorescence

After anesthesia, using 0.9% saline containing 0.1M PB to perfuse the ascending aorta with, sampled were placed in a 4% PFA solution. Then, samples were sunk into 30% sucrose for 5 days, considering dehydration as the brain tissue was removed. Then the brain tissue containing the ACC (bregma +2.2 to +0.5 mm) was sectioned coronally (thickness, 25–35 m) by a cryosectioning device (Leica CM3050S, Wetzlar, Germany). After that, the sections were washed three times for 5 min.

To perform immunohistochemistry, the sections were treated with 5% donkey serum for 1 h at room temperature before incubation with primary antibodies. The tissue sections were then incubated with primary antibodies overnight at 4 °C and washed three times with PBS, followed by incubation with secondary antibodies for 1 h at room temperature.

For the ordinary staining, sections were incubated with single primary antibodies. Incubation with anti-p-RIP1 antibody (1:100, Affinity, Cincinnati, OH, USA), anti-P-RIP3 antibody (1:100, Affinity, Cincinnati, OH, USA), anti-P-MLKL antibody (1:100, Affinity, Cincinnati, OH, USA), anti-RIP1 antibody (1:200, Proteintech, Rosemont, IL, USA), anti-RIP3 antibody (1:100, LifeSpanBioSciences, Seattle, WA, USA), and anti-MLKL antibody (1:100, LifeSpanBioSciences, Seattle, WA, USA) was performed overnight at 4 °C.

To conduct the double or triple staining, sections were incubated with mixed primary antibodies from different species, i.e., incubation was performed with anti-p-RIP1 antibody plus either anti-NeuN (neuronal marker, 1:200; CST, Danvers, MA, USA), anti-parvalbumin antibody (1:500, NOVUS, Littleton, CO, USA), anti-GFAP (astrocyte marker, 1:400; CST), or anti-Iba1 (microglia marker, 1:500; Abcam, Cambridge, UK). Incubation with anti-TNF-α antibody (1:100, Novus, Littleton, CO, USA), anti-parvalbumin antibody, and anti-P-RIP3 antibody or anti-P-MLKL antibody was also performed. The sections were then incubated with donkey anti-Goat IgG H and L conjugated with alexa fluor^®^ 647 (1:400, Abcam, Cambridge, UK), donkey anti-mouse IgG (H + L) conjugated with alexa fluor^®^ 488 (1:400, Abcam, Cambridge, UK), and donkey anti-rabbit IgG (H + L) conjugated with alexa fluor^®^ 555 (1:400, Abcam, Cambridge, UK) for 1 h at room temperature (24–26 °C) after three washes with PBS (10 min each).

Finally, using a CCD point camera (Leica DFC350FX/DMIRB, Heidelberg, Germany) and a fluorescence microscope connected to Leica-IM50 software, the images were captured. Fiji ImageJ2 (National Institutes of Health, Bethesda, MD, USA) or NIS element was used to measure signal intensity. To confirm the specificity of immunostaining with the primary antibody, negative control sections were incubated without primary antibodies.

### 4.5. Western Blotting

Rats were anesthetized and decapitated, and their ACC tissues were quickly removed (0.5–2.2 mm anterior to the bregma in the coronal position) using an anatomical microscope (areas Cg1 and Cg2 in Figure 1A). The brain samples were ultrasonically lysed in RIPA lysis buffer (Beyotime, Shanghai, China), and protease inhibitors (Roche, Mannheim, Germany) were added. The tissue samples were centrifuged at 12,000× *g* for 20 min at 4 °C before target proteins were quantified. Gel electrophoresis (SDS-PAGE) was used to separate proteins, which were then electro-transferred to PVDF membranes (Millipore, Billerica, MA, USA). To seal it at room temperature, 5% skimmed milk was used to for almost 1 h, then end up placing the membranes in p-RIP1 antibody (1:1000, Affinity, Cincinnati, OH, USA), anti-p-RIP3 antibody (1:1000, CST, Danvers, MA, USA), anti-p-MLKL antibody (1:1000, Affinity, Cincinnati, OH, USA), anti-RIP1 antibody (1:1000, Novus, Littleton, CO, USA), anti-RIP3 antibody (1:1000, Novus, Littleton, CO, USA), anti-MLKL antibody (1:1000, Millipore, Billerica, MA, USA), and anti-TNF-α antibody (1:1000, Abcam, Cambridge, UK) overnight in the refrigerator at 4 °C. The samples were then incubated with donkey anti-mouse or anti-rabbit secondary antibodies (1:10,000, Abcam, Cambridge, UK), which were conjugated with HRP. GAPDH (1:3000, Novus, Littleton, CO, USA) was used as a load control comparison strip. The detection of target protein bands using enhanced chemiluminescence (Bio Rad, Hercules, CA, USA) and the Tanon-5200 chemiluminescence imaging system (Tanon Science and Technology, Shanghai, China) was used for imaging. ImageJ imaging analysis Software (NIH, Bethesda, MD, USA) was used to quantify the protein level relative to the level of GAPDH.

### 4.6. Bioinformatic Tools

All expression profiling data analyzed in this study were downloaded from GENEEXPRESSION OMNIBUS (GEO, http://www.ncbi.nlm.nih.gov/geo (accessed on 16 July 2023)). Data from GEO series GSE212311 and GSE228065 were analyzed using Gene Set Enrichment Analysis (GSEA). Genes in the ACC that were differentially expressed between the neuropathic pain models (CCI or SNI) and controls were subjected to GO analysis using the R 4.2.1 and edgeR 3.38.1 packages (https://github.com/ (accessed on 16 July 2023)). The threshold of |logFC| > 1&FDR < 0.05 was used to screen differential genes. Heat and volcano maps were drawn using the R ggplot2 2.2.1 package (https://github.com/ (accessed on 16 July 2023)).

### 4.7. Intra-ACC Drug Application

As described previously [6], stereotactic surgery was conducted in accordance with the rat brain atlas. A stainless steel catheter with a stainless steel cap was inserted into the contralateral ACC and secured with acryl tooth cement. The stereotaxic coordinates for the ACC injection site relative to the bregma were: anteroposterior (AP) +1.5 mm, mediolateral (ML) 0.5 mm, and dorsoventral (DV) −2.5 mm. TNF-α neutralizing antibody (20 μg/mL, 10 μL, R&D Systems, Minneapolis, MN, USA) was slowly injected into the ACC over a period of 5–10 min. The control group was administered a normal IgG control antibody (200 μg/mL, 10 μL, R&D Systems). The TNF-α neutralizing antibody or normal IgG control antibody was administered daily for 8 days, starting from the day before the SNI procedure was performed.

### 4.8. Transfection of siRNA In Vivo

Two kinds of siRNAs were used: (1) disorganizing rat MLKL mRNA (Si-MLKL; Ribobio, Guangzhou, China) to silence MLKL transcription, and (2) scramble siRNA (Si-negative control; Ribobio, Guangzhou, China). These siRNAs can be directly dissolved and used for injection. The cholesterol in its molecular structure was modified (the lipophilicity group) to improve its affinity to the cell membrane. We then dissolved 500 pmol of MLKL siRNA and 500 pmol of scramble siRNA in 5 μL of RNase-free water. After mixing for 5 min, the in vivo-siRNA mixture was injected into the contralateral ACC for 24 h before the SNI procedure was performed. The MLKL siRNA sequences were: sense, 5′-GGAACAACGGAGUAUAUAAdTdT-3′; antisense, 3′-dTdTCCUUGUUGCCUCAUAUAUU-5′.

### 4.9. Supernova

A stereotaxic injection procedure was performed as previously described [5]. For injections into the ACC, mice were anesthetized with 50 mg/kg sodium pentobarbital, and their heads were fixed with a stereotaxic frame. For sparsely labeled ACC neurons, the adeno-associated virus (AAV)-based Supernova vector set consisting of AAV-PTRE-tight-NLS-Cre-WPRE (AAV-PTRE-Cre, Obio Technology Shanghai Corp., Shanghai, China) and pAAV-hSyn-DIO-tTA-P2A-mScarlet-WPRE (AAV-hSyn-DIO-tTA-mScarlet; Obio Technology Shanghai Corp) were injected into the ACC area using a microdispensing pump at the coordinates AP +1.5 mm, ML 0.5 mm, and DV −2.5 mm from the bregma. SNI surgery was conducted at 2 weeks post-viral transfection. The brains were dissected 3 weeks later.

To quantify dendritic spine density, images were acquired with a Nikon C2 (Nikon Eclipse Ni-E) by using a 60× (oil) objective (NA 1.4) with a 2× optical zoom. Z stacks were acquired in a step-size set. Dendritic spine density was quantified using the NIS Element viewer 5.21 by measuring a distance of at least 20 mm along a dendritic branch before counting the number of dendritic spines.

### 4.10. Statistical Analysis

Statistical analysis of immunohistochemical results was performed as described previously [5,6]. GraphPad Prism 6.0 Software was used for multiple or non-duplicate *t*-tests and one-way or two-way ANOVA to determine differences between groups. To test behavioral data, a Dunn’s multiple comparison test or non-parametric two-way ANOVA was performed, followed by a Friedman test. In all cases, results were presented as the mean ± SEM. *p*-values of <0.05 were considered indicative of statistical significance.

## 5. Conclusions

Neuropathic pain is a persistent pain that is treated less effectively than nociceptive pain, especially in the elderly [74]. Exploring the pathogenesis of neuropathic pain provides important guidance for solving clinical treatment problems. The data provided in this study strongly suggest that upregulated TNF-α in the ACC after peripheral nerve injury disinhibits the ACC by inducing PV-IN necroptosis and possibly facilitating spinal nociceptive transmission through descending cortico-spinal pathways. With the aim of elucidating the cortico-spinal mechanism of chronic pain, our future work will explore the involvement of the ACC TNF-α-necroptotic pathway in neural network structures, neuroinflammation, and nociceptive information transmission in the spinal dorsal horn.

## Figures and Tables

**Table 1:** Percentage of ACC necroptosis-associated proteins co-located with cell markers in rats following SNI.

Cell Markers Proteins	NeuN	PV	GFAP	Iba1
RIP1	20.30 ± 1.64%	17.69 ± 2.87%	76.17 ± 1.89%	13.69 ± 1.71%
P-RIP1	79.48 ± 3.48%	70.54 ± 3.15%	12.71 ± 3.65%	15.25 ± 2.35%
RIP3	84.32 ± 3.63%	82.42 ± 2.22%	14.64 ± 1.85%	7.17 ± 0.74%
P-RIP3	83.60 ± 1.24%	73.92 ± 5.58%	29.26 ± 2.40%	8.17 ± 0.91%
MLKL	85.36 ± 2.84%	79.62 ± 0.87%	11.8 ± 1.6%	6.71 ± 0.27%
P-MLKL	93.58 ± 0.36%	84.83 ± 5.51%	5.61 ± 0.41%	4.71 ± 0.48%

NeuN: neuronal marker; PV: PV-INs marker; GFAP: astrocyte marker; Iba1: microglia marker.

## Data Availability

All the necessary data are included within the article. Further data will be shared upon request.

## Supplementary Materials

The following supporting information can be downloaded at: https://www.mdpi.com/article/10.3390/ijms242015454/s1.
