# Supplementary material for: Activation of the TNF-α-Necroptosis Pathway in Parvalbumin-Expressing Interneurons of the Anterior Cingulate Cortex Contributes to Neuropathic Pain
Source: Int J Mol Sci. 2023 Oct 22;24(20):15454. doi: 10.3390/ijms242015454 (PMC10607712; doi:10.3390/ijms242015454)
Supplement: Supplementary file 1 [file ijms-24-15454-s001.zip › ijms-2621620-supplementary.pdf]

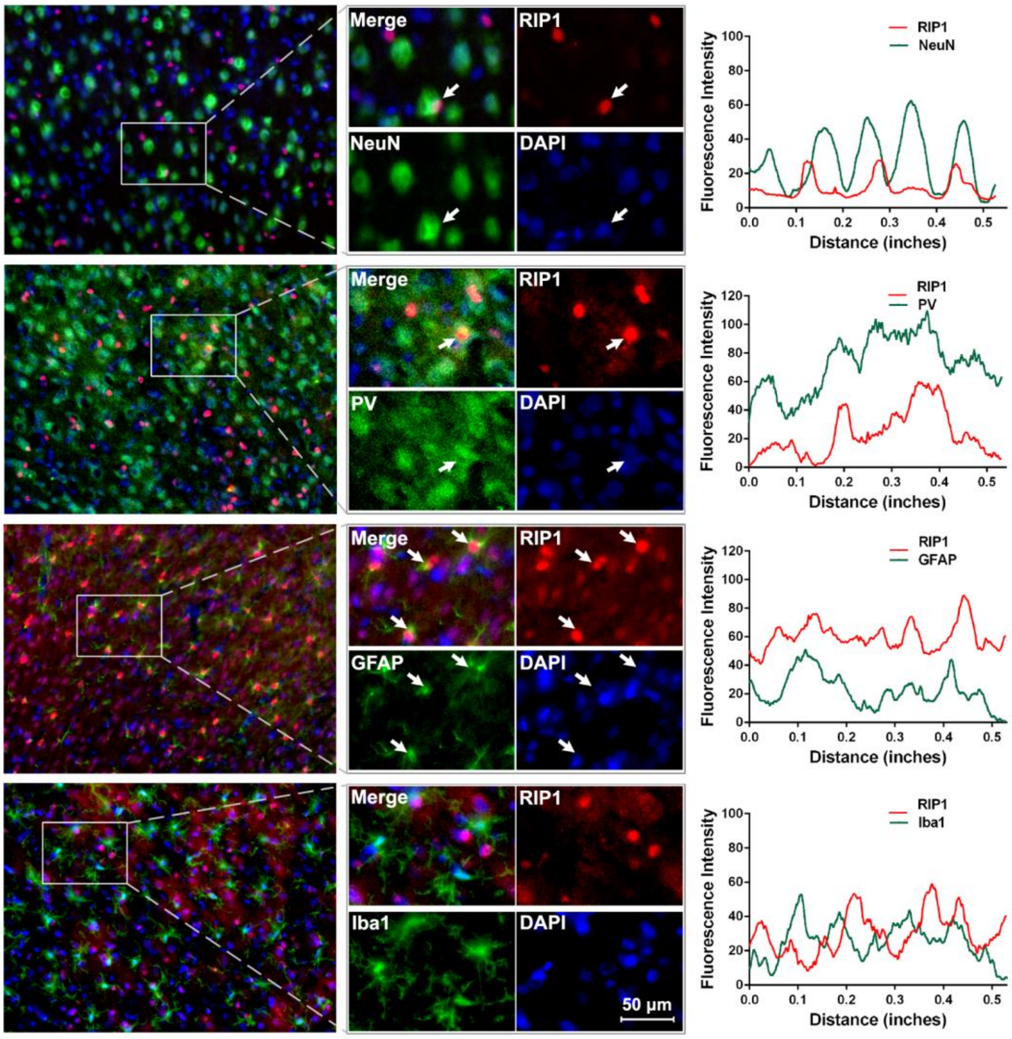

Supplementary Figure S1. Cellular localization of necroptosis-associated protein RIP1 in the ACCs of rats subjected to SNI Representative double staining showing the overlap (yellow) of RIP1 (red) with NeuN (green), PV (green), and GFAP (green), but not with Iba1 (green), on postoperative day 7. Enlarged and color-split images of areas enclosed in white boxes are shown in the middle. White arrows indicate co-localization (yellow). Blue fluorescence corresponds to DAPI. Scale bar = 50 μm. The fluorescence intensity curves for red and green from boxed areas are shown on the right side of each group.

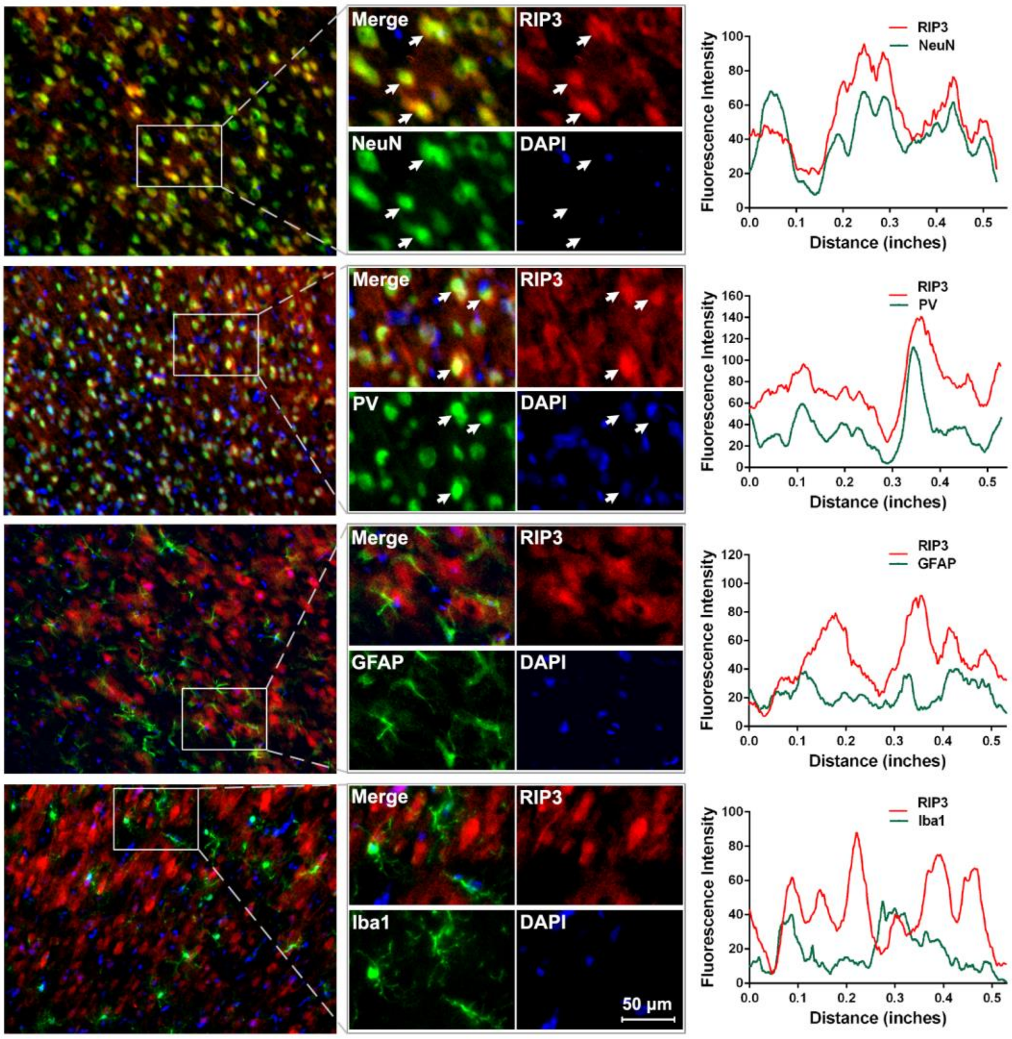

Supplementary Figure S2. Cellular localization of necroptosis-associated protein RIP3 in the ACCs of rats subjected to SNI Representative double staining showing the overlap (yellow) of RIP3 (red) with NeuN (green), and PV (green), but not with GFAP (green) or Iba1 (green), on postoperative day 7. Enlarged and color-split images of areas enclosed inwhite boxes are shown in the middle. White arrows indicate co-localization (yellow). Blue fluorescence corresponds to DAPI.Scale bar = 50 μm. The fluorescence intensity curves for red and green from boxed areas are shown on the right side of each group.

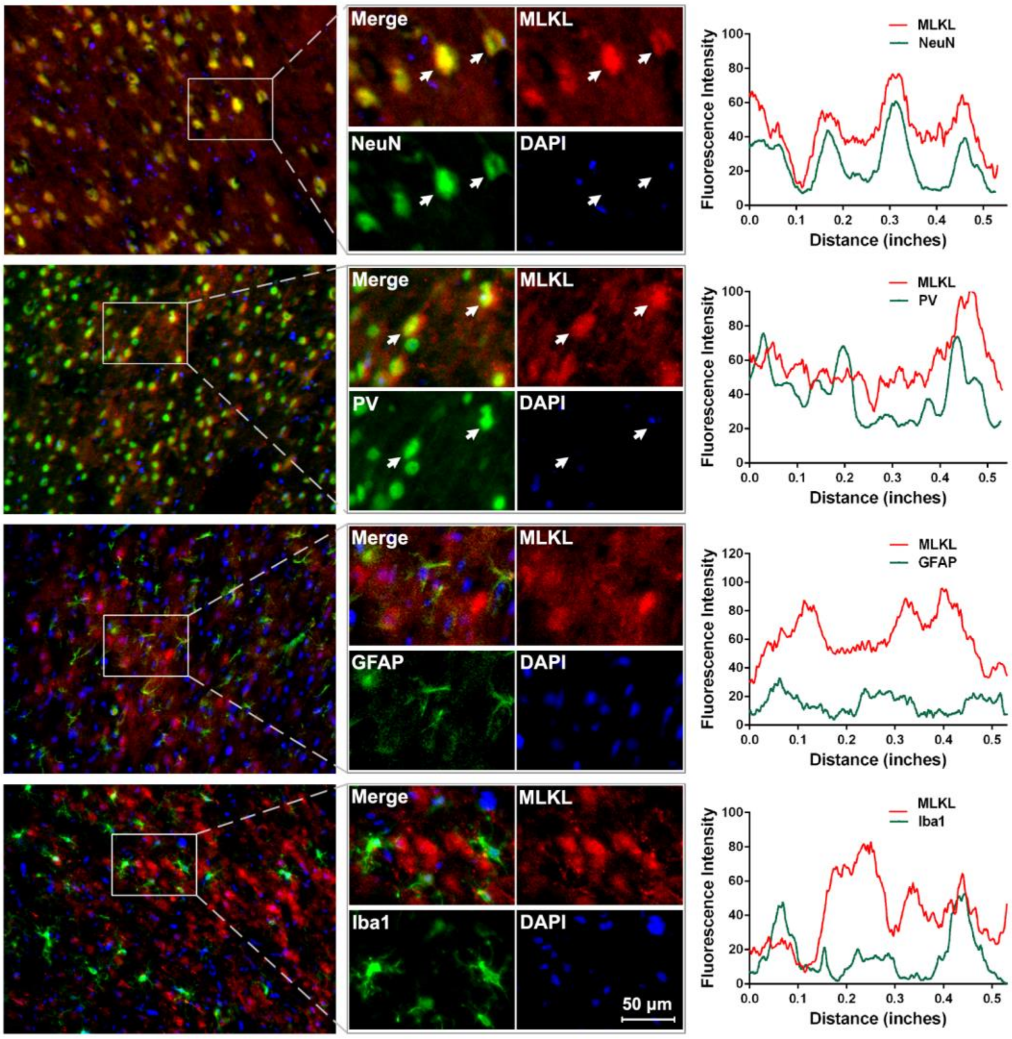

Supplementary Figure S3. Cellular localization of necroptosis-associated protein MLKL in the ACCs of rats subjected to SNI Representative double staining showing the overlap (yellow) of MLKL (red) with NeuN (green), and PV (green), but not with GFAP (green) or Iba1 (green), on postoperative day 7. Enlarged and color-split images of areas enclosed in white boxes are shown in the middle. White arrows indicate co-localization (yellow). Blue fluorescence corresponds to DAPI. Scale bar = 50 μm. The fluorescence intensity curves for red and green from boxed areas are shown on the right side of each group.
